# Computational prediction of inter-species relationships through omics data analysis and machine learning

**DOI:** 10.1186/s12859-018-2388-7

**Published:** 2018-11-20

**Authors:** Diogo Manuel Carvalho Leite, Xavier Brochet, Grégory Resch, Yok-Ai Que, Aitana Neves, Carlos Peña-Reyes

**Affiliations:** 1 School of Business and Engineering Vaud (HEIG-VD), University of Applied Sciences Western Switzerland (HES-SO), Route. de Cheseaux 1, Yverdon-Les-Bains, 1400 Switzerland; 2 SIB Swiss Institute of Bioinformatics, Lausanne, Switzerland; 3 Department of Fundamental Microbiology, University of Lausanne, Lausanne, 1015 Switzerland; 4 Department of Intensive Care Medicine, Bern University Hospital (Inselspital), Freiburgstrasse, Bern, 3010 Switzerland

**Keywords:** Health, Machine learning, Phage-therapy, Supervised learning

## Abstract

**Background:**

Antibiotic resistance and its rapid dissemination around the world threaten the efficacy of currently-used medical treatments and call for novel, innovative approaches to manage multi-drug resistant infections. Phage therapy, i.e., the use of viruses (phages) to specifically infect and kill bacteria during their life cycle, is one of the most promising alternatives to antibiotics. It is based on the correct matching between a target pathogenic bacteria and the therapeutic phage. Nevertheless, correctly matching them is a major challenge. Currently, there is no systematic method to efficiently predict whether phage-bacterium interactions exist and these pairs must be empirically tested in laboratory. Herein, we present our approach for developing a computational model able to predict whether a given phage-bacterium pair can interact based on their genome.

**Results:**

Based on public data from GenBank and phagesDB.org, we collected more than a thousand positive phage-bacterium interactions with their complete genomes. In addition, we generated putative negative (i.e., non-interacting) pairs. We extracted, from the collected genomes, a set of informative features based on the distribution of predictive protein-protein interactions and on their primary structure (e.g. amino-acid frequency, molecular weight and chemical composition of each protein). With these features, we generated multiple candidate datasets to train our algorithms. On this base, we built predictive models exhibiting predictive performance of around 90% in terms of F1-score, sensitivity, specificity, and accuracy, obtained on the test set with 10-fold cross-validation.

**Conclusion:**

These promising results reinforce the hypothesis that machine learning techniques may produce highly-predictive models accelerating the search of interacting phage-bacteria pairs.

## Background

Nowadays, the most-used therapeutic method to treat bacterial infections is the use of antibiotics. However, in recent years, this technique had to face resistance difficulties due to their overconsumption, which threatens medical progress [1]. The increase in resistance makes it harder to fight bacterial infections, that is why alternative methods are required in the near future. The research required to discover new molecules, fueling novel antibiotics, in pharmaceutical laboratories usually implies long time, intensive work, and huge financial effort in comparison with the operating time before the occurrence of resistance. Phage-therapy is one of the most promising re-emergent therapies, consisting in the use of viruses, called bacteriophages, to infect and kill pathogenic bacteria along their life cycle with the aim of curing the infections they cause [2]. These viruses have cohabited and evolved with bacteria, which, along the time, controlled the epidemics, bacterial population and, have contributed to their genetic exchanges since already billions of years. Phages or bacteriophages have the advantage to be extremely strain-specific and do not have a major impact on the commensal flora. The selection of a phage needs to be carefully done due to the fact that some of them can be used for a bacterial treatment but may also drive a horizontal gene transfer contributing to phage resistance.

The first experiments of phage-therapy started at the beginning of the 20^th^ century, when bacteriophages were discovered [3]. In the middle of that century, due to the antibiotics exploration, this therapy was set aside in western countries. Unfortunately, the overexploitation of antibiotics (human health, animal, agriculture,...) has allowed bacteria to develop resistances and, nowadays, the research of new antibiotic molecules is often longer than the time it takes some bacteria to adapt, causing these antibiotics to lose their effect. The concept of phage therapy involves correctly matching a bacterium and a phage able to interact with it. Currently, searching for these pairs is done experimentally in laboratories by means of infection tests [4], process that may take several days of labor. Many positive interactions have been uncovered using these tests revealing, for example, that highly phage-sensitive bacteria get infected by phages with both narrow and broad-host range, whereas highly phage-resistant bacteria are only infected by broad-range phages [5]. There is an increasing number of studies focused on how phages can infect bacteria [6, 7] and on the defense mechanisms developed by bacteria against phage invasion [8]. Receptor-binding proteins (RBPs) in phages are able to recognize and bind specifically to receptors on the surface of the bacterium. These bacterial receptors have been experimentally identified in some cases and shown to generally involve both proteins and cell-wall glycopolymers [9]. When the phage is bound and connected with the bacterial host, it injects its genome inside the bacterial cytoplasm. Only the phage genome can enter in a target bacteria.

Phages can be classified in two categories according to the way their genome develops inside the bacteria: (1) Tempered phages that follow the lysogenic cycle, whose genome can integrate with the bacterium DNA, becoming a prophage that follows bacterial cell division. When the cell is under stress (e.g., cell damage), the prophage becomes active and initiate the “lytic cycle”. (2) Virulent, or lytic, phages, whose replication begins immediately after injecting their genome, resulting in bacterial wall disruption and destruction due to holins and lysins activity. Lytic phages are more suitable for phage therapy. A recent machine-learning approach, called PHACTS (Phage Classification Tool Set) [10], is able to automatically identify the type of life cycle of a phage based on its protein sequence.

Bacteria and phage constantly adapt their defense and attack mechanisms [6, 7]. One method used by bacteria to prevent phage attack is to render their receptors unrecognizable for the phage through mutations on them. Another mechanism is to hide the receptors’ binding regions with capsules as physical barriers [8]. They may also develop the ability to block phage DNA injection when a second phage is trying to infect them [9]. Some bacteria are also able to detect genetically-encoded sites that could be targeted by a restriction-modification system which cuts stranger DNA at specific recognition sites (e.g., the CRISPR/Cas system, evolved by bacteria, is a kind of prokaryotic immune system that confers resistance to a phage). Some bacteria choose to suicide to prevent their replication and to avoid any type of reproduction (abortive infection system [7]). Finally, phages can be defeated by bacteria through phage assembly interference, where bacteria encode phage-inducible chromosomal islands capable of negatively interacting with the assembly of the phage [11].

Thus, the host range of a phage not only depends on its own attack mechanisms: receptor-binding and lysins, but also on the bacterial defense mechanisms. Naturally, phages found in man-made and/or natural environments co-evolve quickly with their bacterial target. In consequence, the infectivity of a phage may differ from one host bacterial species to another and even from one strain to another of the same bacterial species [12]. Currently, the host range of a phage is determined by means of infection tests [4] usually based on spot assays or, more recently, on methods such as microfluidic-PCR or PhageFish [13, 14]. All these methods, depending on the number of bacterial hosts tested, may require several days of laboratory work.

As already mentioned, phage-therapy is one of the most promising alternatives to fight against the emergence of multi-resistant bacteria. Usually, phage-therapy is performed by using cocktails of different phages able to kill a specific population of bacteria [2]. These cocktails contain both lytic phages able to lyse the bacteria from outside [15] and temperate phages that add extra genes to the bacteria making them to lose their resistance, allowing thus to treat the patient with normal antibiotics [16, 17]. Several preclinical and veterinary trials [1] have shown good results but, unfortunately, phage-therapy still requires having a completely-characterized phage library as well as methods able to quickly detect a potential phage collection for a specific bacterial strain. A recent work by Volddby Larsen et al. [13] proposes a computational approach and a companion bioinformatic tool named HostPhinder, that deals partially with this goal as it predicts the bacterial host of a given phage based on its genome, by computing its similarity with the genomes of other phages with known host. There exist other approaches able to detect a phage-host range. For instance, Coelho et al. [18] propose a method based on PPIs, and Edwards et al. [14] propose a method based on sequence similarity resorting to techniques like Blast. Computational modeling-based approaches like PHAST (PHAge Search Tool) [19] are able to detect if a given bacteria contains a prophage using genomic information and BLAST matching to a phage-specific sequence. All these methods are based on sequence similarity to make their predictions.

This is where our approach steps in, as it might be used to automatically identify, from a phage library, a number of candidate phages able to infect a given pathogenic bacterium based mainly, or solely, on their genomes. To achieve this, we combine state-of-the-art techniques from machine-learning and bioinformatics with genomic data and the ever-growing information about phage-bacteria interactions. We conceived, explored and implemented an original approach, based on supervised modeling, to predict if a given phage-bacterium pair would interact. To build such predictive models based exclusively on genomic information, one of the biggest challenges resides in the, so-called, feature engineering. It consists in, first extracting informative features that capture essential properties of the phage and the bacterium. Then, in further selecting a subset of these features that allow the models to obtain the best predictive results.

## Methods

### Creation of the dataset

To create our training dataset, we extracted phage-bacterium pairs that have been annotated in public databases as exhibiting (positive) interactions. In order to complete the training dataset, we generated putative non-interacting (i.e., negative) pairs, since the public databases do not clearly annotate the absence of interaction. Two public databases were used to collect the complete genomes of all bacteria and phages: PhagesDB [20] and GenBank [21], consulted in February 2016. We compiled 1064 phage sequences—79 from GenBank and 986 from PhageDB—as well as 42 host bacteria sequences, extracted from GenBank. It results, thus, in a total of 1064 positive phage-bacterium interactions.

#### Phage sequences

As mentioned above, we obtained a first set of 986 complete phage genome sequences from PhageDB [20]. From this data we performed gene prediction, so as to retrieve coding-DNA and protein sequences, using GeneMarkS [22]. We retrieved a second set of 79 phage sequences from GenBank accessed through the Entrez Nucleotide service [23, 24] which provides directly the genome, coding-DNA, and protein sequences. (The query ‘phage [Title] and complete genome’ was used to obtain the information for each phage.)

#### Bacterial sequences

We parsed the annotation of each phage to obtain its bacterial host, by extracting it from the fields ‘Isolation Host’ and ‘host’, respectively, in PhagesDb and GenBank. The genome, coding-DNA, and protein sequences, for each bacterium, were extracted with the Entrez Nucleotide service, using the query ‘name of bacteria [ORGN] AND ‘complete genome’. All phages whose bacterial host was unknown or did not have a complete sequence were removed.

#### Positive interactions

As already mentioned, the initial positive dataset contains 1064 phage-bacterium pairs with annotated interactions. Among them, 915 correspond to the same bacterial host (i.e., *M. Smegmatis*). Unfortunately, such over-representation of a single bacterium in the dataset may have a negative effect on the pertinence of the models obtained during the learning phase. In effect, a model based only on the presence or absence of *M. Smegmatis* to predict interactions. To palliate this effect, we grouped the interactions by bacterial families based on the NCBI’s taxonomy database [25] and further balanced their presence in the training dataset by means of oversampling—or replacement-sampling—a technique used to balance datasets containing classes with very different number of instances [26]. We applied this technique to our positive interactions dataset using two steps: (1) grouping the interactions considering their bacterial families, we obtained 19 families with, in average, two bacteria. (2) replicating the interactions of each family as many times as necessary to ensure that it is represented around 300 times—excepting for the family containing *M. Smegmatis*. E.g, the family “Alteromonadaceae” which contains a total of four interactions (2 bacteria, each with two interactions) is replicated 75 times. This approach allows reducing the over-representation of *M. Smegmatis*. The oversampled dataset is composed by 6’517 interactions of which 915 involve *M. Smegmatis*, representing 14% of the interactions—against 86% for the original data.

#### Negative interactions

Ideally, a negative dataset should contain phage-bacteria pairs that have been shown, experimentally, to not interact. Unfortunately, to the best of our knowledge, no data source provides such an information. For this reason, we created a set of putative negative interactions using all the phages and bacteria from the positive dataset. From all the possible phage-bacterium pairs, a given pair would be considered as not-interacting if it satisfies two conditions: (1) it does not exist in the positive set and (2) the bacterium belongs to a different species than that of the phage’s known host. Although, these criteria do not warrant that a given pair won’t interact at all, it will select pairs that are not known to physically interact and that are not likely to do it, considering the high specificity of phages to one bacterial species, even to specific strains within a species [27, 28]. This approach results in more than 43’000 putative negative interactions pairs. As before, in order to improve the relevance of the models extracted from the data, we decided to maintain the same number of negative pairs for each bacterial species than in the positive dataset. Whenever possible, these pairs are randomly selected from the putative negative set. In the case where the number of available negative pairs is not enough, some of them are repeated.

### Feature extraction: Protein-Protein Interactions

The interactions between a phage and a bacterium are, in principle, mainly due to the interactions between their encoded proteins. So, one can expect protein-protein interactions (PPIs) to contain relevant information for predicting phage-bacterium interactions. In this section, we present the methodology used to extract two different sets of features, based on PPIs [18], that constitute the base for our candidate training datasets. In our database bacterial genomes encode, in average, for 3’417 proteins, whereas an average phage expresses 74 proteins, resulting in 74×3417≈2.5×10^5^ PPIs for an average phage-bacterium pair. Note, nevertheless, that the number of PPIs may be (very) different from one phage-bacterium pair to another. In consequence, during the feature extraction stage, it is necessary to apply some kind of post-processing to make them comparable and easily exploitable by the machine learning algorithms. We extracted two kinds of features from these PPIs: domain-domain interaction scores and protein primary structure information, as explained below.

#### Domain-domain interaction scores

A domain is defined as a structural or functional subunit of a protein [29, 30]. Often, a PPI involves one or more bindings between pairs of their constituting domains. DOMINE [31] is a database of known and predicted protein domain interactions—or domain-domain interactions (DDIs). It contains DDIs observed in PDB crystal structures as well as those predicted by several computational approaches. In DOMINE, all DDIs are obtained using Pfam HMM profiles for protein domain definitions [32], to detect them in our proteins we used the HMMER API [32, 33]. Each DDI is evaluated by a quality score that represents the predicted quality of the interaction, computed by several algorithms. The cumulated interaction score of a PPI is then calculated as the sum of all its DDIs. Our database contains more than 2.2×10^5^ proteins (from both bacteria and phages) with more than 3.5×10^5^ domains. Using the scoring procedure described before, we obtain a vector of PPI scores for each phage-bacterium pair. To deal with the different vector lengths of these scores, we transformed them into a vector of frequencies (a histogram of PPI-scores) in order to obtain vectors of the same size. Doing so, we explored two parameters: (1) using normalized or absolute frequency, and (2) predefining the size of the histogram bins (SB) or their number (NB). We produced thus, four different kinds of datasets from the DDI scores, as described in Table 1.

**Table 1:** DDI-score-based datasets

Histogram’s bin generation	Normalized	Values	Abbreviation
Fixed number of bins	Yes	5, 10, 15, 30, 50	NBN sets
	No	5, 10, 15, 30, 50	NB sets
Fixed-size bins	Yes	1, 5, 10, 15, 20	SBN sets
	No	1×10^−6^, 2.5×10^−6^, 5×10^−6^	SB sets

Different configurations were used to generate 18 datasets based on the frequency distribution of domain-domain interaction scores. There exist four types of datasets according to (1) whether the histogram’s bins are defined with fixed size or fixed number and (2) whether or not the score frequencies are absolute or normalized values

#### Protein primary structure information

A second set of features is based on the physicochemical properties of the proteins of each PPI [34–37]. Using the sequence of each protein, we extracted the following 27 features: 21 representing the frequency of each amino-acid—i.e. the 20 amino-acids plus one for unknown amino-acids; five other features corresponding to the abundance of selected chemical elements composing the proteins (i.e. Carbon, Hydrogen, Nitrogen, Oxygen, and Sulfur) [38] and, finally, the molecular weight of the protein. So, for each protein-protein interaction, we have 54 features. As already mentioned, each phage-bacterium pair has, in average, more than 2.5×10^5^ PPI scores, representing an extremely high dimensionality. In order to reduce it, we calculated, for each phage-bacterium pair, the mean and the standard deviation for the features across all its PPIs. At the end, each phage-bacterium pair is represented by 108 features, 54 mean values and 54 standard-deviation values.

In summary, 19 candidate datasets were created based on the two extracted types of features: 18 based on DDIs and one based on primary structure information, dubbed chemical composition (CH). A set of 13’034 phage-bacterium pairs, comprising an equal number of positive and negative interactions, was selected for feature extraction and to generate the datasets. Ten percent (10%) of the data was removed and used to create a stratified test set. For each bacteria family, the same proportion of interactions were taken into consideration for creating the train and the test sets. We then used a machine learning-based process, as described in next section, to investigate them and select the most informative datasets in order to constitute the final training dataset.

### Predictive modeling and machine learning

We constructed a predictive model able to evaluate if a phage-bacterium pair can interact or not. Four machine-learning modeling techniques were explored in order to find their best configuration parameters as well as to identify the datasets that more consistently allow for the best prediction scores. These methods are: K-Nearest Neighbors (K-NN) [39], Random Forests (RF) [40], Support Vector Machines (SVM) [26], and Artificial Neural Networks (ANN) [41].

The modeling process was performed in two phases: exploration and refinement. The exploration phase allowed us to test multiple algorithm configurations performing a grid search with multiple parameter values for each modeling technique and for each dataset. The refinement phase extended the exploration of the number of neurons for ANNs on some selected datasets. Indeed, we noticed that their performance increased with such a number and required for further investigation. All along the process we used 10-fold cross-validation [26] in order to prevent model over-fitting and to optimize model selection. The predictive performance was assessed by using several metrics: accuracy, f-score, specificity, and sensitivity.

## Results

### Exploratory phase

**Fig. 1:**
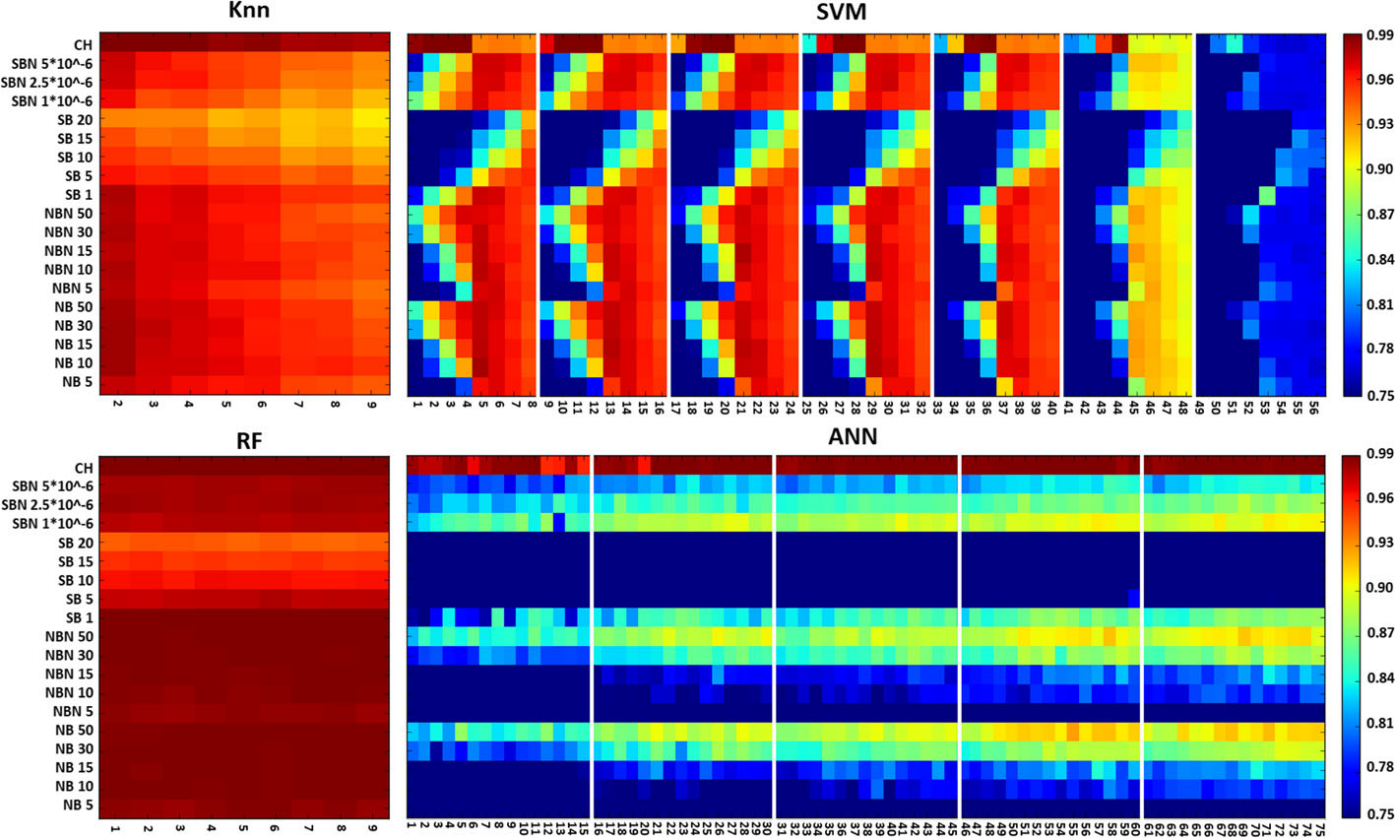
Heatmaps resuming the F1 scores obtained during the exploratory phase. Each heatmap represents the results obtained by all the configurations for each method: K-NN (top left), RF (bottom left), SVM (top right), and NN (bottom left). The lines in the heatmap correspond to the different datasets and the columns correspond to the different configurations. The vertical white lines indicate the change of one parameter value: number of neurons for ANN and penalty factor for SVM

Given the large space of parameters explored for each algorithm, as well as the large number of datasets on which they were tested, we visualized all the results with heatmaps in order to better analyze them. Figure 1 shows, as an example, the heatmap of the F1-score results obtained in the exploratory phase. Note that during all the process we considered four performance metrics—i.e., sensitivity, specificity, accuracy, and F1 score to make the decisions.

At first sight, it seems that RF and K-NN attain excellent predictive performances while SVM and ANN are less predictive. However, such good results ask for special scrutiny and analysis:

- K-NN: it bases its predictions on the similarity of each case (i.e., interaction) with its closest neighbors. As our dataset includes repetitions to reduce the over-representation problem of a bacterium, K-NN is fooled by this redundancy and obtains false high performances. This is clearly illustrated in Fig. 1 where it attains more than 96% of F1-score for almost any combination of dataset and k-values.
- RF: It seems clear, from the very-high performance figures, often superior to 98% (see Fig. 1), that RF is also not performing well with repeating data. Indeed, as already reported in the literature, RF is closely related to K-NN and both can be viewed as weighted neighborhood schemes that make predictions by looking at the “neighborhood” of the target point [42].
- SVM: From the results, we can observe that the best results are obtained on the datasets CH, SBN 1×10^−6^, NBN50, and NB50 when using small values for momentum and penalty. For those parameter configurations, the F1-score shown in Fig. 1 takes on values bigger than 85%.
- ANN: Looking at the results in Fig. 1, the main conclusion we can make is that the more neurones are in the hidden layer, the better are the results. This is particularly visible for the four datasets mentioned above. One may obtain more than 88% of F-1 score with six neurones in hidden layer.

Considering these results, we decided to maintain four datasets—SBN 1×10^−6^, NBN50, NB50 and CH for further experiments. Note that the performances obtained on the CH dataset are so high that one could consider it as over-fitting. We decided, however, to keep it and validate such hypothesis on the final test set (i.e., the one never used for training nor validation).

### Refinement phase

**Fig. 2:**
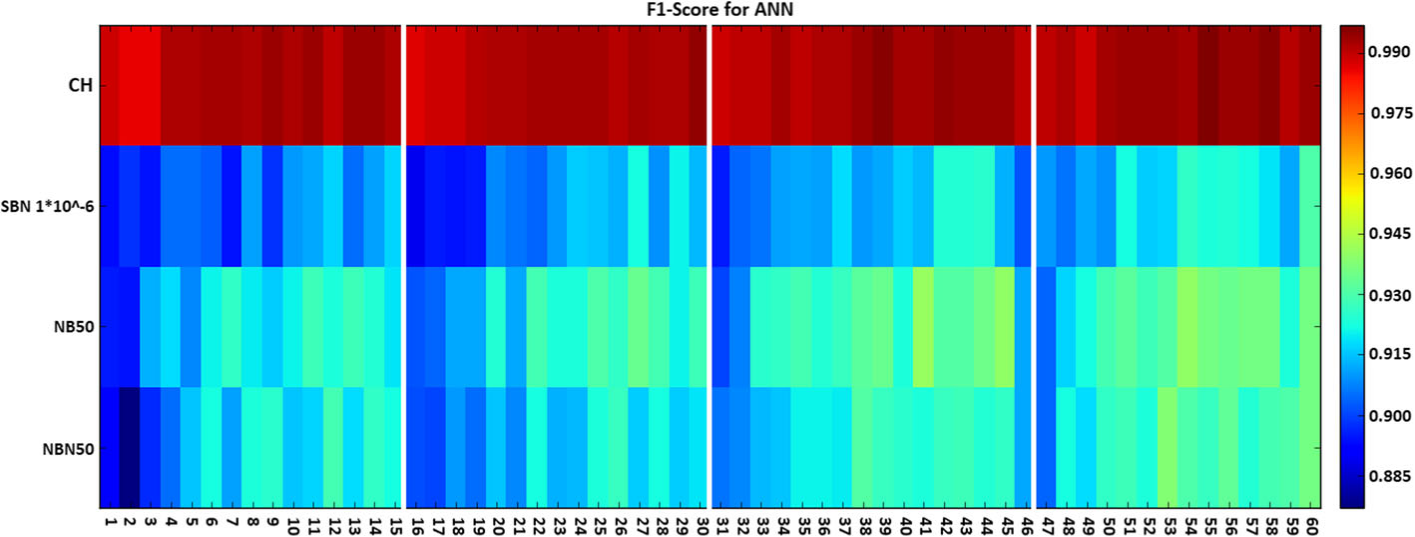
F1-score results obtained in the refinement phase. Each line represents a different dataset, while the columns correspond to the different combinations of parameter values detailed in Table 2. The change on the number of neurons is represented by the vertical white lines

From the previous analysis we can conclude that the number of hidden neurons in ANN is the only parameter that deserves further investigation. Figure 2 shows the F1-score results obtained for the additional configurations described in Table 2. Although the performance continued to increase with the number of neurons in the hidden layer, it stagnated with around 9 or 10 neurons. The F1-score reached 93% for the best configuration with the selected datasets. Note that the refinement phase was performed only on ANN as it is the only method whose results in the exploring phase are not conclusive enough. As already discussed, K-NN and RF overfitted the data and do not deserve more exploration. On the other hand, observing the SVM results, the parameter values allowing for the best performances are clear making it unnecessary to explore more configurations.

**Table 2:** Configurations used along the machine-learning algorithm in both exploratory and refinement modeling phases

			Modeling phase	
		Parameters	Exploratory	Refinement
Method	K-NN	K	{1,2,3,4,5,6,7,8,9}	
	RF	N-trees	{10^2, 10^3, 10^4}	
		L-size	{2,3,4}	
	SVM	Penalty	{10^4, 10^3,... 10^-2}	
		Momentum	{10^-4, 10^-3,..., 10^4}	
	ANN	N-neurones	{2,3,4,5,6}	{7,8,9,10}
		Epochs	{10,25,50,75,100}	{10,25,50,75,100}
		Momentum	{0.1,0.4,0.7}	{0.1,0.4,0.7}
Datasets			All 19 sets	SB1E-6, NBN50, NB50, CH

Based on the analysis of all the results, we selected as the best configuration the use of an ANN with 9 neurons in the hidden layer, trained during 50 epochs with 0.1 of momentum value and 0.01% as learning rate. The 10 models obtained by cross-validation during the refinement phase were evaluated on the test set (i.e., a set that was kept apart since the beginning and was never used during the modeling phases). Table 3 summarizes the results obtained on such tests. As expected, the performance figures on the test set are lower than those on the validation sets for all selected datasets. Nevertheless the performance loss is relatively small—e.g., accuracy loss ranges from around 4.7% for SBN 1×10^−6^ to around 1.1% for CH.

**Table 3:** Summary of the results obtained by the selected modeling approach (i.e., ANN with 9 neurones in the hidden layer) on both validation and test conditions.

DataSet	Accuracy		F-Score		Sensitivity		Specificity	
	Val.	Test	Val.	Test	Val.	Test	Val.	Test
CH	99,0%	97,9%	99,0%	97,0%	99,9%	97,5%	98,6%	98,3%
SBN1E-6	90,4%	85,7%	90,6%	86,2%	90,5%	85,4%	90,9%	86,3%
NB50	91,4%	88,2%	91,7%	88.5%	91,1%	88,6%	92.1%	87.7%
NBN50	92,4%	89,8%	92,5%	90,1%	93,6%	90.7%	91,3%	88.8%

## Discussion

The emergence of antibiotic-resistant bacteria is a serious threat for medicine and health care. Phage-therapy, i.e., the use of viruses (phages) to fight bacteria, is a promising alternative to heal patients suffering of antibiotic-resistant infections. The main challenge for such therapy is to rapidly and effectively find the correct phage (or a handful of them) able to attack the target bacterium. To address this challenge, we explored the use of machine learning techniques to build models able to predict if a given phage-bacterium pair would interact, based on the genome sequences of both organisms. In order to train the models, our approach first builds a number of training datasets based on informative features obtained from the genomic data. For this purpose, we concentrated our analysis on protein-protein interactions and extracted, for each PPI, two types of features: one based on domain-domain interactions and another on chemical composition. Subsequently, we explored several machine-learning techniques on all these datasets in order to select a configuration (i.e., an algorithm and its parameter values) producing the most predictive models. The results obtained, with accuracy values ranging from 86% to almost 90% on test data, are encouraging.

Note that it is hard to make a direct comparison between the present work and our early approach. Indeed, as already explained, an oversampling was applied to our data so as to palliate the problem of the M. Segmatis bacterium being over-represented in the original dataset. As a consequence of this modification, K-NN and RF became unusable because of the repeats. The classification performances obtained on this new dataset are similar to those obtained previously, meaning that the other methods were able to adapt to the new data distribution. Another major difference with the previous results is the classification performance obtained on the CH dataset. This is due to the fact that this dataset is no longer based on principal component analysis, as this technique didn’t convey enough information to be predictive, but it is now calculated as the mean and the standard deviation of each feature.

Thanks to the experience acquired, we have identified the following issues that we should address in the future.

- A first improvement should relate with the number and the diversity of the phage-bacterium pairs included in the database. Indeed, our positive dataset contains only 1’064 positive interactions, from which 915 were based on the same bacterial species (M. Segmatis) creating a serious bias on the data and limiting the predictive power of the models. The dataset should be enriched with more interaction pairs involving other bacteria. In the same sense, only a few pairs of our current data correspond to different strains of the same bacterial species. In consequence, it is not currently possible to make predictions at the strain level. We plan to add more strain-specific interactions to our database;
- We will also consider two alternative strategies to avoid repeating interaction pairs in the dataset. In a first approach, we may under-sample the positive interactions containing *M. Smegmatis* so as to generate several small-but-balanced, training datasets. Then, obtaining classifiers for each dataset and, finally, combining them in a single ensemble-classifier. A second alternative would be to assign weights to each phage-bacterium pair inversely proportional to the relative frequency of its bacterium. In that way, those pairs containing *M. Smegmatis* would contribute much less to the global error while learning the classifiers;
- Finally, another limitation concerns the current relevance of the features that depend on the DOMINE database, as its last release dates back to 2007.

## Conclusions

In conclusion, the present work showed the potential of using machine-learning methods to predict if a given phage-bacterium pair will interact. The increasing amount of annotated interactions and that of available bacterial and viral genomes, together with advances in the comprehension of phage biology, leads us to think that we will soon have enough information to develop novel *in silico* tools that accurately predict phage-bacterium interactions. Such tools would contribute to the development of personalized therapies against bacterial infections and will reduce the time required to search for such a treatment.

Some ideas we are planning to further investigate include (1) performing “one-class learning” based only on validated positive interactions, (2) using reinforcement-learning, which is based on a reward system used alongside the training process, to drive the identification of genetic code relevant for phage-bacterium interaction, and (3) applying sequence-oriented machine learning techniques to learn directly from the genetic sequences instead of, or in addition to, extracted features.

## Abbreviations

ANN: Artificial neural network
CH: CHemical composition
DDI: Domain-domain interaction
KNN: K-nearest neighbors
NB: Number of bins
NBN: Number of bins normalized
PPI: Protein-protein interaction
RF: Random forest
SB: Size of bins
SBN: Size of bins normalized
SVM: Support vector machine
